# Metallosis After Oxinium Total Knee Arthroplasty in a Patient With Rheumatoid Arthritis: A Case Report

**DOI:** 10.7759/cureus.34541

**Published:** 2023-02-02

**Authors:** Stalin Cañizares, Gabriela Carolina Carrera Barriga, Fabiana Valencia Jarrín, Carlos Daniel Poveda Freire

**Affiliations:** 1 General Surgery, Universidad San Francisco de Quito, Quito, ECU; 2 Trauma and Orthopaedics, Universidad San Francisco de Quito, Quito, ECU; 3 Trauma and Orthopaedics, Universidad San Fracisco de Quito, Quito, ECU

**Keywords:** case report, rheumatoid arthritis, oxinium, total knee arthroplasty, metallosis

## Abstract

Metallosis is a late uncommon complication of knee arthroplasties due to prosthetic loosening or component displacement. Oxinium prosthesis used to have components that attempted and proved to decrease prosthetic wear and consequent metallosis in the past. However, new studies showed that a combination of a shallow anterior tab snap-fit locking mechanism and thin dovetail lips make it susceptible to polyethylene dislocation and prosthesis loosening. The following case report show metallosis development in a 69-year-old female patient with a 20-year history of stage IV left gonarthrosis (Kellgren and Lawrence classification) who underwent a total knee arthroplasty (TKA) with a high-flex PS Genesis II prosthesis (Smith & Nephew, Hertfordshire, UK). We discuss the role of the material and her rheumatoid arthritis background in orthopedic mechanical failure. It is crucial that designers focus on the improvement of locking mechanisms and polyethylene properties.

## Introduction

Metallosis is a late uncommon complication of joint arthroplasties relatively more prevalent in high-wear joint replacement such as knees and hips [1,2]. Nevertheless, knee metallosis is rare compared to the hip joint because polyethylene is used as a bearing surface [1]. Fractures, prosthetic loosening, and component displacement constitute orthopedic hardware complications that may be etiologically related to metallosis [2].

Metallosis is a rare complication characterized by the infiltration of periprosthetic soft tissue and bone by metallic debris [2] mainly due to early degeneration of the polyethylene component [1]. Platicosis, which is the deposition of small particles of polyethylene in the joint space, constitutes the primary event that will later result in profuse polyethylene wear and early prosthesis loosening [1]. An additional contact between the cement and the femoral implant accelerates the wear and creates methacrylate particles that act as abrasive [3]. Once a metal-on-metal articulation is formed, metal debris causes inflammation by one of three mechanisms: type IV hypersensitivity, direct toxic effect, and particle-induced synovitis [4]. This hypersensitivity reaction begins with the combination of metal ions (like haptens) with large carrier protein molecules causing massive cytokine release. This promotes lymphocytic, histiocytic, and giant cell infiltration that causes synovial hyperplasia and the development of a black material in the synovium [2,4,5]. As a result, chronic synovitis, osteoclastic bone resorption, tissue necrosis, and the development of pseudo tumours may occur [2,5]. Implant loosening happens when histiocytes, which phagocyte metallic particles, release inflammatory cytokines and induce fibrosis [4]. Moreover, necrosis of the liver and spleen and even systemic toxicity may develop if lymphatic or vascular invasion exists [5].

Most people are asymptomatic until polyethylene wear is significant enough to cause pain, rigidity, instability, mechanical clicking, and large joint effusions on examination [5]. Some authors describe a gradual worsening knee pain associated with a noticeable rash [5]. The first-line imaging modality is a simple radiography is needed. There are three typical appearances: the “cloud sign” characterized by periprosthetic amorphous densities, the “metal line sign” as a thin hyperdense suprasellar line, and the “bubble sign” as curvilinear radio intensities outlining the entire joint [2,6]. Slight anterior subluxation of polyethylene liner on lateral radiographs is an early indicator of locking mechanism failure [7]. Narrowing or asymmetry of the joint space in comparison to previous imaging is another potential indicator [7]. Other complementary tests include serum laboratory studies (white blood cell count, C-reactive protein, erythrocyte sedimentation rate) and synovial fluid analysis (culture, gram stain, cell count, white blood cell count, alpha defensin). Intraoperative cultures are also needed to rule out periprosthetic infection [6].

## Case presentation

**Figure 1:**
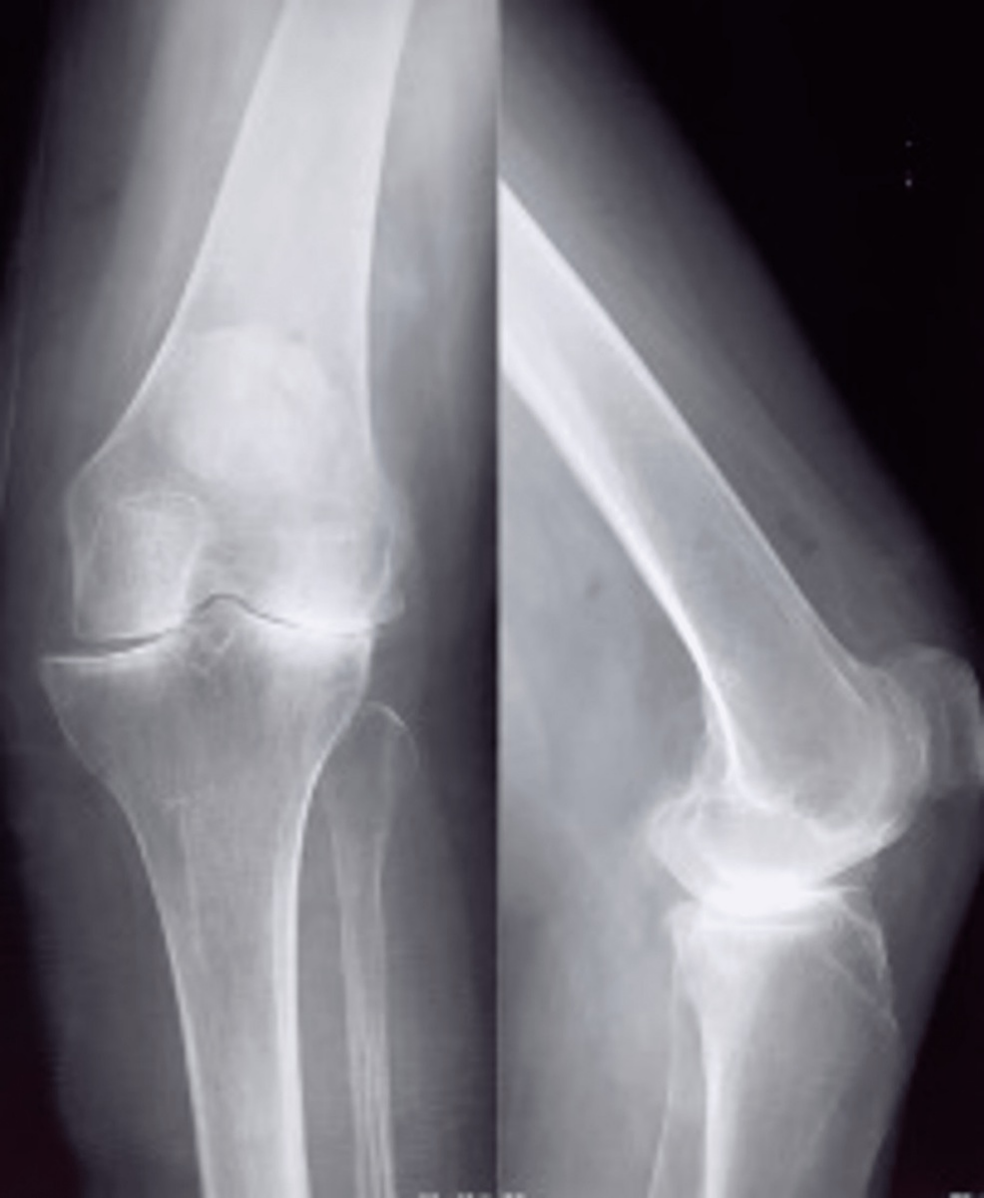
X-ray images of the affected knee Stage IV left gonarthrosis (Kellgren and Lawrence classification) previous to the Total Knee Arthroplasty

**Figure 2:**
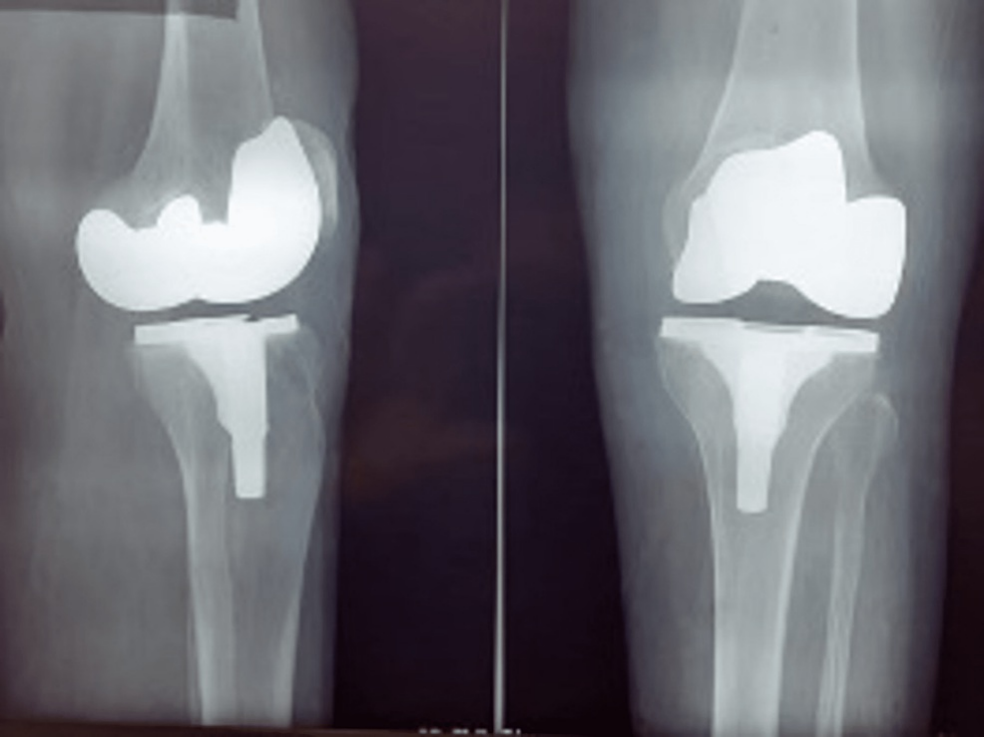
Postoperative control two years after Total Knee Arthroplasty

A 69-year-old female patient with a 20-year history of stage IV left gonarthrosis (Kellgren and Lawrence classification) (Figure 1) underwent a total knee arthroplasty (TKA) with a high-flex PS Genesis II prosthesis (Smith & Nephew, Hertfordshire, UK: femoral component 4, tibial component 2, no patellar change, fixed insert 2x9 mm) in 2016 (Figure 2). Her past medical history was significant for rheumatoid arthritis, arterial hypertension, type 2 diabetes, and a previous TKA in her right leg in 2018 (Attune, DePuy Synthes, Jhonson-Jhonson).

**Figure 3:**
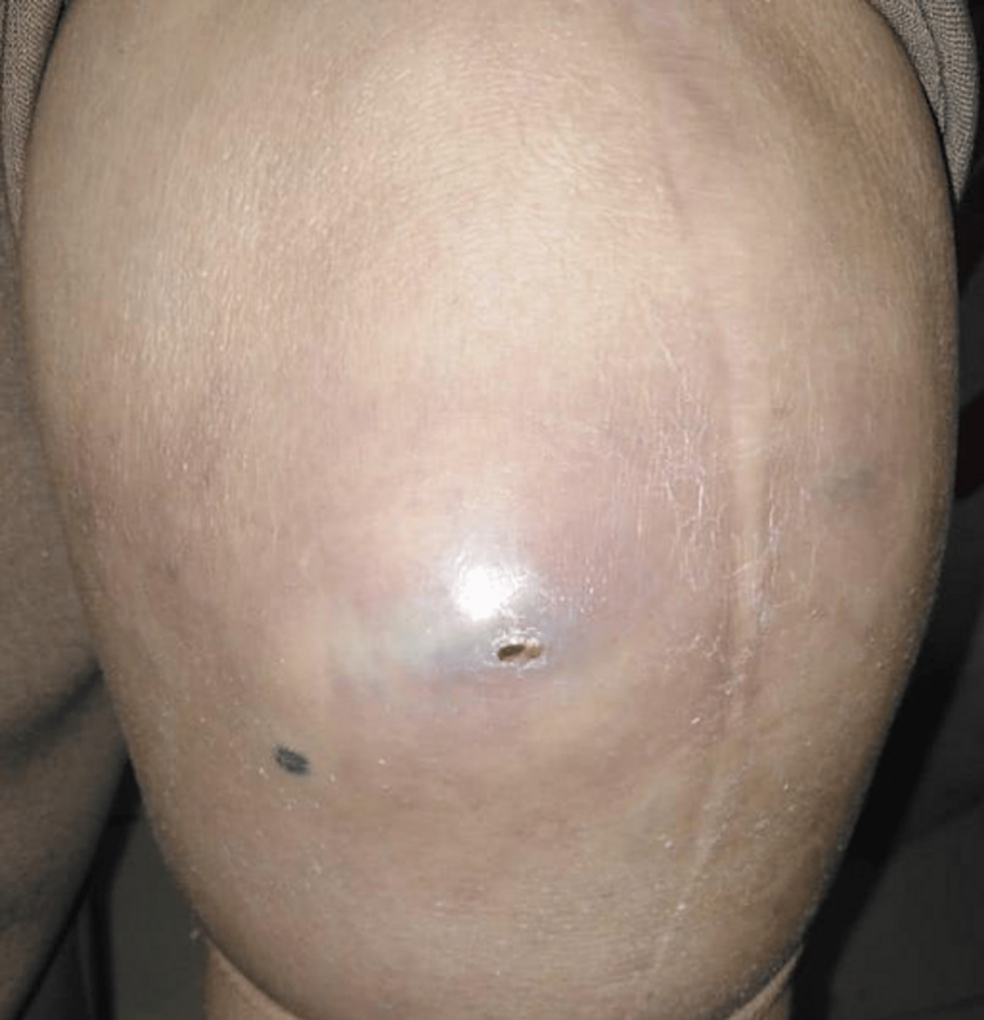
Swollen left knee of the patient Swollen left knee articulation showing an internal anterior protrusion corresponding to the polyethylene dislocation four years after Total Knee Arthroplasty.

**Figure 4:**
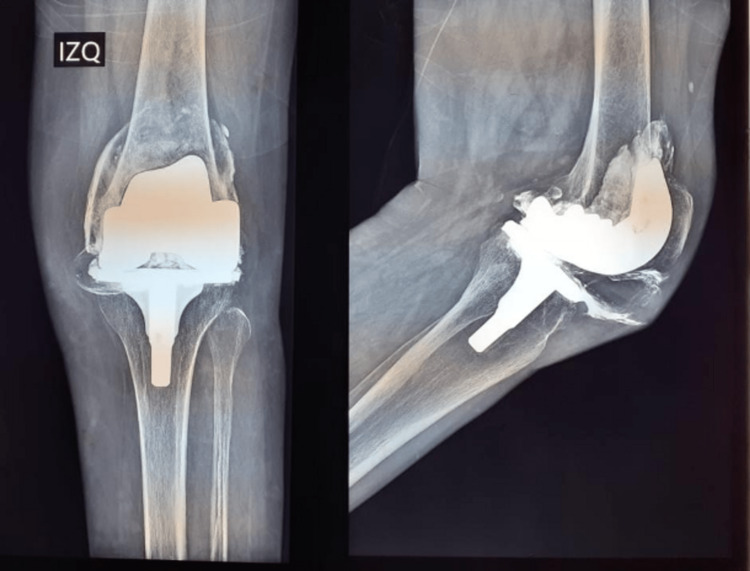
Radiograph images of the swollen knee Thin hyperdense line outlining a portion of the joint capsule which probably corresponded to the “metal line sign” observed metal-induced synovitis.

Initially, she had a 18º valgus, a KSS (Knee Society Score) of 3, and a Function score of 45. Her postoperative course was uneventful until October 2019 when he stumbled and fell on her left knee causing pain and increasing disability. Since her symptoms did not improve, the patient came for clinical assessment after one month. At physical examination, swelling and a growing mass in the internal anterior angle were observed (Figure 3). Her initial workup revealed normal inflammatory markers in serum and a negative articular puncture for infection. Radiographs revealed a narrowing of the joint space and a thin hyperdense line outlining a portion of the joint capsule which probably corresponded to the “metal line sign” observed metal-induced synovitis (Figure 4).

**Figure 5:**
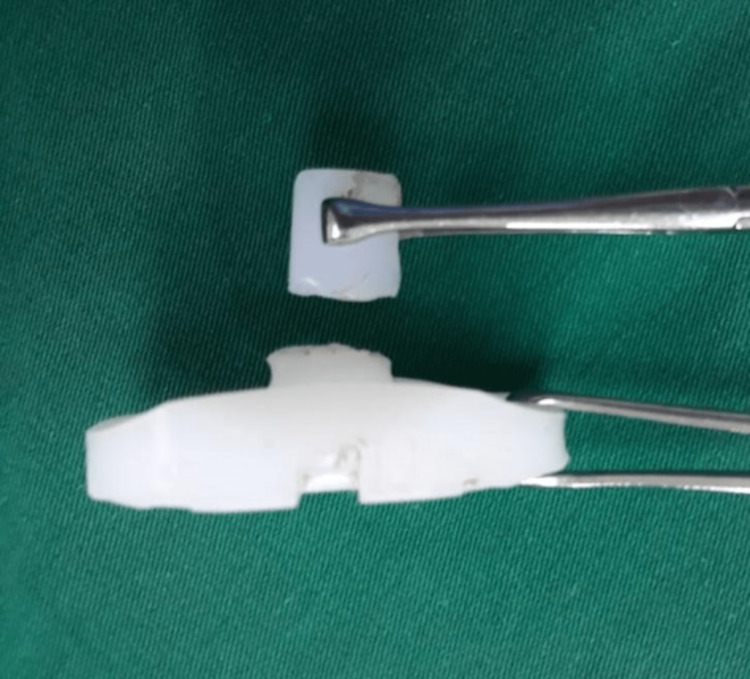
Intraoperative finding in revision surgery Post detachment from the tibial plate.

**Figure 6:**
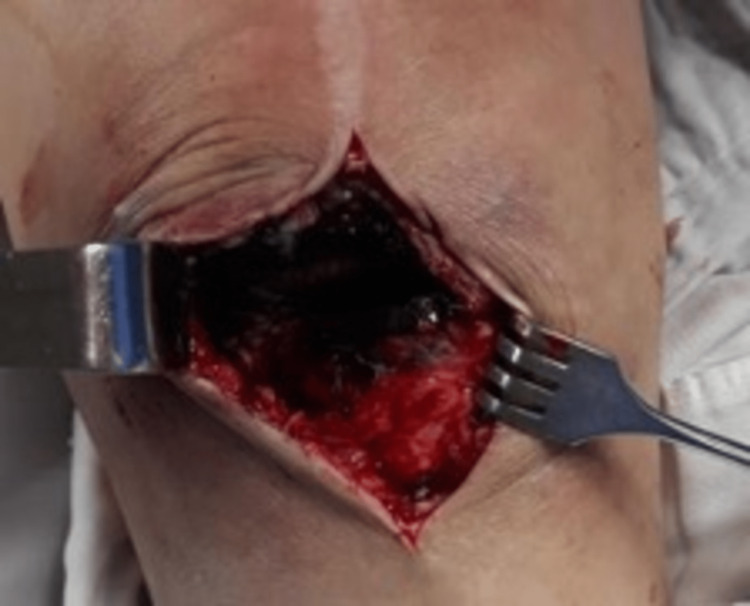
Intraoperative finding in revision surgery Diffuse dark black synovial thickening was seen.

At revision surgery, polyethylene liner dislocation towards the internal anterior knee compartment and a profuse metallosis was observed. Skin integrity was intact. Femoral and tibial components were well fixed with no evidence of loosening. However, delamination of the femoral component in the inferior external region was noted. Post was detached from the tibial plate making the whole rollback (post/cam) mechanism obsolete (Figure 5). A synovectomy was performed demonstrating diffuse dark black synovial thickening (Figure 6). Debridement of soft tissue impacted by insert dislocation, medial and lateral gutters and all damaged tissue down to healthy appearing tissue was performed. Samples were sent for histopathological assessment and culture. Insert was replaced by a new one of the same size. The culture was negative for infection and histopathology reported granulation tissue, neutrophilic and lymphocytic infiltration, and black pigment within macrophages. The patient was discharged after one week and post-operative controls were performed to assess soft tissue and the potential risk for loosening recurrence. Second-time revision surgery was planned for the future. However, the overall prognosis was good, and no further surgery was expected to occur. Now, the patient has not suffered from any episode as before and therefore, has not required additional treatment.

## Discussion

Metallosis is a complication following arthroplasty that is primarily manifested by pain, swelling, dislocation, and instability [8]. An early diagnosis and proper management are needed to avoid further complications [8]. Usually, the breakdown and loosening of components are inevitable because of progressive metal and polyethylene degradation [6]. However, deterioration of the opposite compartment and polyethylene tibial insert wear may occur early causing metal-on-metal articulation, aseptic loosening, and metallosis [9]. A major cause of loosening is attributed to the failure of the polyethylene locking mechanism which impairs the control of the interface micromotion and causes a dissociation of the polyethylene insert from the tibial base plate and an unintended articulation of metal components [10]. This failure might be explained by one of three scenarios: surgical technique, patient’s unfortunate events, and prosthesis design decision. 

During surgery, an incorrect bearing insertion and a conservative resection of the bone can eventually lead to polyethylene liner dissociation [4,10]. In fact, dissociation of a fixed bearing insert after TKA, which is a rare event, has been significantly related to intraoperative misplacement of the insert resulting in ligament imbalance, excessive polyethylene wear, and locking mechanism failure [3]. A mini-subvastus approach limits surgical exposure causing soft tissue impingement or incomplete seating of the tibial insert [3]. Patients' unfortunate events, especially trauma, establish an important cause of acute gross dislocation [8]. Repetitive micromotion between the bearing and base plate from intense physical activity, particularly in patients with high BMI, can also cause slight wearing of the polyethylene and varus deformity that later potentiates load on the medial aspect of the knee resulting in more wear on the polyethylene and final gross dislocation [4,10]. If no definite trauma history, ligament laxity is the usual trigger of late insert dissociation [3]. Prosthesis design is a strong determinant of patient outcome. Loosening is more common in mobile-bearing TKA due to poor component alignment, improper soft-tissue balance, retained soft tissue, and the development of osteophytes [3,10]. Impingement of an osteophyte on the posterior femoral condyle with the knee in deep flexion is a possibility even in fixed-bearing TKA [5]. Although rare in fixed-bearing TKA, it may happen with cruciate-retaining type prostheses [3]. Other mechanical risk factors include flat designs, increased rotational freedom, and dislodgement of the modular tibial liner which usually allows stability after implanting final components [4,7,10]. Some designs use a metal clip to hold the polyethylene liner to the tibial base plate to provide more stability. Nevertheless, it has been reported that the breakage of this component is a potential trigger for prosthesis loosening and post-operative knee metallosis [9]. 

In our patient, all components were strongly fixed to the bone and no evident initial rotational malpositioning during surgery was observed. Delamination of the femoral component in the inferior external region and soft tissue laceration by insert dislocation were noted. It is possible that trauma was the initial event that lead to a gross dislocation of the polyethylene liner. Later, continued left knee flexion caused a rupture of the post from the tibial plate. It is possible that this detachment worsens the roll/back mechanism resulting in the development of a metal-on-metal articulation and, eventually, metallosis. It is important to consider that apparently only one month had passed from the fallen to metallosis’ clinical picture.

Our patient received a high-flex posterior-stabilized (PS) Genesis II TKA (Smith & Nephew). There are reports that about 98.7% of ceramic-surfaced oxidized zirconium femoral components survived and functioned for up to seven years [10]. However, two reports have described tibial insert dissociation after high-flex posterior-stabilized (PS) Genesis II TKA (Smith & Nephew, Memphis, Tenn) due to early displacement, incomplete seating of the tibial insert at surgery, limitation of exposure, trauma, and tibial post damage [3]. These articles report that this design has been proposed to be more prone to insert dissociation because of a combination of a shallow anterior tab snap-fit locking mechanism and thin dovetail lips [3]. When the knee flexes, and if the insert was not sealed properly into the tibial metal tray, the downward force on the posterior half of the polyethylene insert would cause anterior lift-off of the tibial component. This initial lift-off completes the dissociation of the insert from the tibial component, thanks to the shallowness of the anterior tab. Furthermore, the thin dovetail lips are more susceptible to damage causing deformations that result in micromotion which leads to the eventual failure of the locking mechanism [3]. Paradoxically, its peripheral dovetail lock is the tightest on the market and maintains structural integrity under excessive loads which limits micromotion and the associated risk of osteolysis [10]. 

This Oxinium Genesis II prosthesis has three major components: a femoral made of zirconium and niobium (oxidized surface into zirconium oxide ceramic), a patellar made of polyethylene, and a modular tibial one with a UHMWP (ultrahigh molecular weight polyethylene) insert in a titanium-aluminium-vanadium alloy base plate [1]. Hypersensitivity to metal is common in up to 15% of the population [9]. Metallic implants may trigger a type IV hypersensitivity reaction in which implant debris causes macrophage activation with subsequent release of interleukin (IL)-1b, tumour necrosis factor (TNF), IL-6, and IL-8 activating osteoclasts leading to cutaneous eczematous eruptions, device failure, chronic inflammation and pain, loosening of joint prostheses, osteolysis, metallosis, excessive periprosthetic fibrosis, and muscular necrosis [1,10]. The finding of metallosis and extensive lymphocytic and plasma cell infiltration around the metal debris support the association between metal hypersensitivity, osteolysis, and aseptic loosening [4]. Oxidised zirconium is better than other prosthesis’ components such as cobalt chromium in resisting roughening, frictional behaviour, biocompatibility, and reducing the wear of the polyethylene under normal and abrasive conditions [1]. Specific cell-mediated response to ultrahigh molecular weight polyethylene (UHMWP) does not appear to play a major role because of their relatively large-sized degradation products [9,10]

Data about rheumatoid arthritis (RA) being a risk factor for prosthetic joint infection and/or mechanical complications is still very limited [4]. A study found that risk factors for mechanical complications due to osteolysis, aseptic loosening, and dislocation were RA, respiratory comorbidity, anemia, any previous infection, use of corticosteroids, and other comorbidities [5]. Mechanical complications were particularly related to osteoporosis which had three major potential issues: perioperative fractures, an increased risk of periprosthetic fracture, and a mechanical failure of ingrown trabecular bone [5]. A deeper association between RA and implant loosening is still unclear because of a possible decreased wear of the implant due to lower physical activity and a less propensity to perform revisions on patients due to comorbidities and fragile bones [5]. In our patient, it is likely that rheumatoid arthritis in association with a series of unfortunate events led to the insert dislocation and the development of metallosis.

## Conclusions

Complete removal of wear metallic particles in case of metallosis is recommended to avoid possible immunological reactions and periprosthetic osteolysis secondary to the release of bone-resorbing cytokine. In our patient, revision surgery and synovectomy were performed to prevent progressive destruction of the entire joint. As all femoral and tibial components were fixed without apparent loosening, only the insert was replaced by a new one of the same size. Debridement of soft tissue impacted by insert dislocation was necessary. And, because of these intraoperative findings, a future second-time revision surgery was planned. Designers should focus on the improvement of locking mechanisms and polyethylene properties. In theory, oxinium components were new alternatives that attempted to decrease prosthetic wear and consequent metallosis. Our report presents a case of dislocation and aseptic loosening resulting in metallosis after the placement of an oxinium prosthesis.
